# S-1 Maintenance Therapy After First-Line Treatment With Nab-Paclitaxel Plus S-1 for Advanced Pancreatic Adenocarcinoma: A Real-World Study

**DOI:** 10.3389/fonc.2022.865404

**Published:** 2022-05-13

**Authors:** Yan Shi, Quanli Han, Huan Yan, Yao Lv, Jing Yuan, Jie Li, Shasha Guan, Zhikuan Wang, Lei Huang, Guanghai Dai

**Affiliations:** ^1^ Department of Oncology, Ruijin Hospital, School of Medicine, Shanghai Jiao Tong University, Shanghai, China; ^2^ Department of Medical Oncology, Chinese People's Liberation Army (PLA) General Hospital, Beijing, China; ^3^ Department of Pathology, Chinese People's Liberation Army (PLA) General Hospital, Beijing, China; ^4^ Medical Center on Aging of Ruijin Hospital, Shanghai Jiao Tong University School of Medicine, Shanghai, China

**Keywords:** maintenance therapy, pancreatic adenocarcinoma, S-1, nab-paclitaxel, survival, safety

## Abstract

**Background:**

In our previous phase II study, nab-paclitaxel plus S-1 (NPS) showed encouraging objective response rate (ORR) as first-line treatment for advanced pancreatic adenocarcinoma (APAC). This study aimed to evaluate the effectiveness and safety of S-1 maintenance after NPS in APAC and to explore factors predicting survival benefits when using S-1 maintenance.

**Methods:**

Between 2014 and 2018 a total of 182 patients with APAC, who were primarily treated with NPS, were included. For patients without progression or with treatment discontinuation due to any reasons within 4 months during NPS treatment, S-1 monotherapy was administrable as maintenance therapy at the physicians’ discretion based on the patients’ preference and performance status. Efficacy and safety of S-1 maintenance were investigated.

**Results:**

In 123 patients without progression within 4 months during NPS treatment, 74 received S-1 maintenance and had median progression-free survival of 9.6 months and median overall survival of 16.7 months. Multivariable analysis showed that in patients receiving S-1 maintenance after first-line NPS therapy, an Eastern Cooperative Oncology Group (ECOG) Performance Status score of 0, non-metastatic disease, and complete or partial response as best response to NPS chemotherapy were independently associated with better survival. The most common all-grade hematological and non-hematological adverse events were neutropenia (82.4%) and peripheral neurotoxicity (66.2%), respectively, and the most common ≥Grade 3 hematological and non-hematological adverse events were neutropenia (40.5%) and peripheral neurotoxicity (6.8%), respectively in patients who received S-1 maintenance.

**Conclusions:**

Our real-world study showed that S-1 maintenance after tumor response or stable disease induced by first-line NPS treatment was effective and well-tolerated for some patients with APAC, which offers a promising alternative treatment strategy with encouraging survival for APAC.

## Introduction

Pancreatic cancer, the majority of which is pancreatic ductal adenocarcinoma (PDAC), accounted for almost as many new deaths (~466,000) as new cases (~496,000) in 2020 due to its poor prognosis and it is the 7^th^ leading cause of cancer mortality in both sexes (1). Advanced PDACs (APACs) with involvement of major arteries (e.g., celiac trunk and superior mesenteric artery) or with metastasis to distant sites, which we previously found to comprise ~64-81% of all PDACs in Europe and the US (2), are often deemed unresectable. Chemotherapy remains the major treatment modality for APAC, and can significantly prolong survival and improve quality of life (3). The prognosis for APACs is extremely poor, the 3-year overall survival (OS) being 2-5% for total patients <60 years and 1-2% for those ≥60 years in Europe and the US, where only slight improvements in survival were observed over time (4).

Growing evidence supports that maintenance therapy offers clinical benefits in pancreatic, colorectal, and lung cancers (5–7). Although the POLO study demonstrated that progression-free survival (PFS) was longer with olaparib maintenance than with placebo (7.4 vs. 3.8 months) among patients with metastatic pancreatic cancer and a germline BRCA mutation, PARP inhibitors were less commonly used than chemotherapy agents in the maintenance setting due to the low proportion of germline BRCA mutation (8). A phase II study showed that induction gemcitabine and S−1 (GS) followed by chemoradiotherapy and S−1 maintenance for locally-advanced disease was feasible, well-tolerated, and highly active, with median survival of 22.9 months and 3-year survival rate of 30.4% (9). Maintenance with capecitabine for metastatic disease treated with first-line FOLFIRINOX was also effective, with median OS (mOS) of 17 months and first PFS of 5 months (10).

Among various chemotherapy regimens for APAC, nano-albumin-bound (nab)-paclitaxel plus S-1 (NPS) is effective under careful toxicology surveillance. As first-line chemotherapy for metastatic disease, it significantly prolonged median time to progression (7.1 vs. 3.6 months) and OS (10.2 vs. 6.0 months) compared to GS in a retrospective study (11). Our previous phase II trial (clinicaltrials.gov registration number: NCT02124317) supported that NPS as first-line treatment with S-1 as optional subsequent maintenance therapy had encouraging objective response rate (ORR; 50.0%) and survival (mOS, 9.4 months; median PFS [mPFS], 5.6 months) and manageable toxicities for APAC, suggesting this strategy as an effective alternative treatment regimen (12).

In this study, we described the patient and tumor characteristics, tumor responses, and safety profiles in APAC patients treated with induction NPS therapy and subsequent S-1 maintenance. We further described the survival and explored the prognostic factors among patients receiving S-1 maintenance both overall and in various stratifications. Our findings suggested that for APAC patients the NPS therapy may not need to be administered until disease progression and may be replaced by S-1 maintenance after objective response or stabilization of disease, which is effective with low toxicity.

## Patients and Methods

### Study Population

This study was based on our previous phase II clinical trial on the NPS regimen (12). Patients with histologically confirmed advanced (locally advanced or metastatic) pancreatic adenocarcinoma, treated with NPS as first-line chemotherapy at Chinese PLA General Hospital between January 2014 and December 2018, were eligible for this study. Patients were primarily given NPS if they were between 18 and 80 years of age, had an Eastern Cooperative Oncology Group (ECOG) Performance Status score of 0 to 1, had adequate bone marrow (leukocyte count ≥3500/mm^3^, neutrophil count ≥1500/mm^3^, platelet count ≥100,000/mm^3^, and hemoglobin level ≥10 g/dL), renal (creatinine level ≥1.5 mg/dL), and liver function (bilirubin level ≤3 mg/dL, and alanine aminotransferase and aspartate transaminase levels ≤3 times the upper limit of normal values), and measurable lesion according to the Response Evaluation Criteria in Solid Tumors (RECIST; version 1.1) (13). The main exclusion criteria were as follows: pathologically diagnosed acinar cell cancer, no completion of 2 cycles of NPS, no imaging assessment after treatment, other cancer diagnosed simultaneously, NPS received in neoadjuvant setting, treatment by radiotherapy or other ablation before first-line NPS, and less than 6 months from adjuvant chemotherapy to first-line chemotherapy. All patients signed written informed consent to receive NPS as first-line chemotherapy and offered their medical records in advance for research analysis.

### Treatment Strategies

Advanced pancreatic cancer patients were primarily treated with NPS as first-line chemotherapy for at least 2 cycles. Nab-paclitaxel was administrated at 240 mg/m^2^ body surface area every 3 weeks and S-1 was given at 80-120 mg/m^2^ body surface area per day on days 1-14 of each 21-day cycle. For patients without progression or with treatment discontinuation due to any reason within 4 months during NPS treatment, S-1 monotherapy with dose as above was allowed to be administrated as maintenance therapy at the physicians’ discretion based on the patients’ preference, ECOG performance status, and on evaluations of efficacy and benefits.

**Figure 1:**
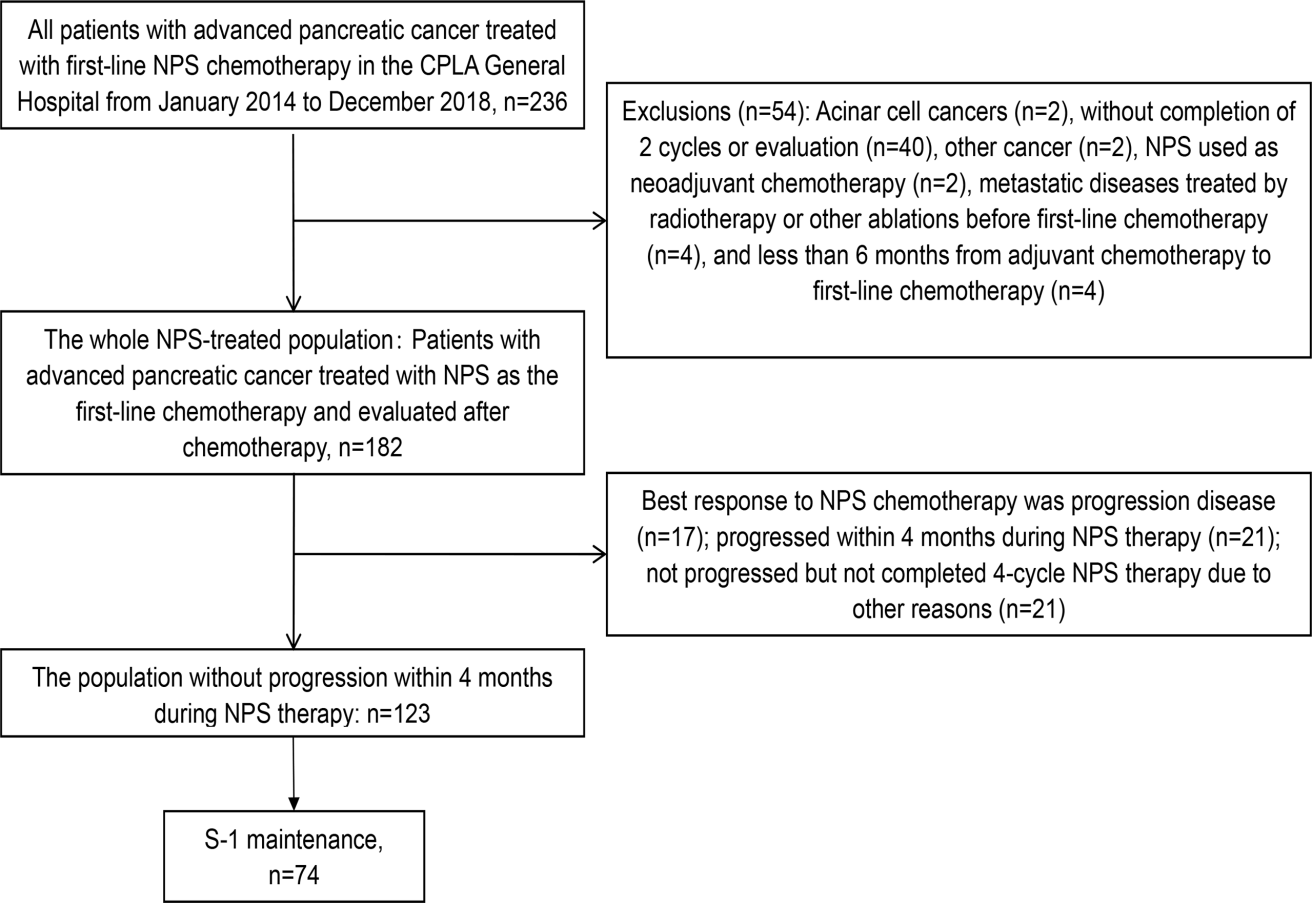
Flowchart of selecting the study population.

A total of 182 patients with APAC who received NPS as first-line chemotherapy followed by evaluation were included in this analysis (**Figure 1**). After excluding cases where best response to NPS chemotherapy was progression disease, where disease progressed within 4 months during NPS treatment, or had no progression but had not completed 4-cycle NPS chemotherapy, a total of 123 eligible patients (including 74 with S-1 maintenance) were followed-up every 2 months until disease progression or death. After disease progression, second-line chemotherapy, clinical trial, or best supportive care was recommended based on tumor burden and patients’ performance status.

### Key Variables Assessments

Cycles of NPS and S-1, changes of tumor biomarkers, ORR, PFS, OS, treatment-related adverse events (all grade and ≥Grade 3; hematological and non-hematological), and other patient and tumor characteristics were measured and recorded. Age and baseline CA19-9 levels were dichotomized based on the median values. Tumor biomarkers and treatment-related adverse events were assessed every cycle of NPS or S-1. Adverse events were classified according to the Common Terminology Criteria for Adverse Events (CTCAE; version 4.0). Patients were evaluated by CT or MRI scan every 2 cycles of NPS or S-1. CA19-9 response was defined as >50% decline from baseline CA19-9 level within 4 cycles of NPS chemotherapy. ORR was evaluated by an independent reviewer according to the RECIST criteria (version 1.1), and partial or complete response or stable disease needed to be confirmed after ≥4 weeks. PFS was calculated from the first day of receiving NPS to disease progression or death, and OS from the first day of receiving NPS to death.

### Statistical Analyses

Survival was calculated using the Kaplan-Meier method and was compared using the log-rank test. Factors associated with survival were explored first using univariable and then multivariable COX proportional hazards regression with hazard ratios (HRs) and corresponding 95% confidence intervals (CIs) calculated. In the main multivariable model, clinicopathologic parameters including S-1 maintenance therapy, age, sex, ECOG performance status, stage, primary tumor location, differentiation grade, number of metastases, baseline CA19-9 levels, and best response to chemotherapy were mutually adjusted for metastatic site available for metastatic diseases only and CA19-9 response evaluated only in patients with elevated baseline CA19-9 levels, that were additionally included into the main model when calculating multivariable-adjusted HRs for them. The proportional hazards assumption was verified both graphically using the log-log plot and analytically using the scaled Schoenfeld residuals test before performing modelling survival analyses (14). Statistical significance was defined as having a *P* value of <0.05. Statistical analyses were performed with the SPSS software (version 19).

## Results

### Baseline Characteristics and Tumor Responses

Among 182 APAC patients receiving NPS chemotherapy, 123 without progression of disease within 4 months during NPS treatment, including 74 with S-1 maintenance therapy, were eligible for analyses (**Figure 1**) and the characteristics of each group are shown in **Table 1**. Regarding tumor responses, 89.8% of patients who received subsequent S-1 maintenance therapy achieved CA19-9 responses, and 77.0% had objective (complete or partial) response as best response to chemotherapy during NPS treatment.

**Table 1:** Baseline characteristics and tumor responses.

Variables	Total patients receiving NPS, n=182	Patients without progression within 4 months during NPS treatment	
Baseline characteristics		Overall, n=123	With S-1 maintenance, n=74
**Age (years)**			
Median (range)	58 (34-78)	58 (34-76)	59 (34-76)
**Sex (%)**			
Male	108 (59.3)	66 (53.7)	37 (50.0)
Female	74 (40.7)	57 (46.3)	37 (50.0)
**ECOG PS score at baseline (%)**			
0	142 (78.0)	96 (78.0)	55 (74.3)
1	40 (22.0)	27 (22.0)	19 (25.7)
**Stage (%)**			
Locally advanced	15 (8.2)	14 (11.4)	9 (12.2)
Metastatic	167 (91.8)	109 (88.6)	65 (87.8)
**Location of primary tumor (%)**			
Head/neck	62 (34.1)	40 (32.5)	26 (35.1)
Body/tail	120 (65.9)	83 (67.5)	48 (64.9)
**Tumor differentiation (%)**			
Well/well-moderately/moderately differentiated	67 (36.8)	51 (41.5)	35 (47.3)
Moderately-poorly/poorly differentiated	115 (63.2)	72 (58.5)	39 (52.7)
**Metastasis site (%)** ^&^			
Liver only	66 (39.5)	35 (32.1)	20 (30.8)
Liver and others	66 (39.5)	45 (41.3)	25 (38.5)
Others except liver	35 (21.0)	29 (26.6)	20 (30.8)
**Number of metastases**			
0-1	92 (50.5)	59 (48.0)	35 (47.3)
2	53 (29.1)	42 (34.1)	27 (36.5)
≥3	37 (20.3)	22 (17.9)	12 (16.2)
**Elevated levels of tumor biomarkers at baseline (%)** ^#^			
Elevated level of CA19-9 only	22 (12.1)	20 (16.7)	16 (22.2)
Elevated levels of CA19-9 and others	134 (73.6)	81 (65.9)	43 (59.7)
Elevated levels of others except CA19-9	23 (12.6)	19 (15.4)	13 (18.1)
**Baseline CA19-9 level**			
Normal	26 (14.3)	22 (17.9)	15 (20.3)
Elevated	156 (85.7)	101 (82.1)	59 (79.3)
**Baseline CA19-9 level**			
<2000 U/mL	94 (51.6)	77 (62.6)	52 (70.3)
≥2000 U/mL	88 (48.4)	46 (37.4)	22 (29.7)
**Cycles of first-line NPS chemotherapy**			
Median (range)	5 (2 - 12)	6 (4 - 12)	6 (4 - 12)
**Tumor responses**			
**>50% decline from baseline CA19-9 level in 4 cycles (%)** ^$^			
Yes	94 (59.1)	79 (77.5)	53 (89.8)
No	65 (40.9)	23 (22.5)	6 (10.2)
**Best response to NPS chemotherapy (%)** ^**^			
CR or PR	98 (53.8)	84 (68.3)	57 (77.0)
SD	67 (36.8)	39 (31.7)	17 (23.0)
PD	17 (9.3)	NA	NA
ORR	53.8%	68.3%	77.0%
**PFS** (months), median (95% CI)	6.0 (5.0, 7.0)	8.0 (7.2, 8.8)	9.6 (8.4,10.8)
**OS** (months), median (95% CI)	11.2 (9.5, 13.0)	14.3 (12.4, 16.2)	16.7 (13.9, 19.5)

^&^Metastasis site was evaluated only in metastatic diseases, the number of which were 167 in the total NPS-treated patients and 109 in the NPS-treated patients without progression within 4 cycles of treatment.

^#^Three patients had normal levels of tumor biomarkers at baseline.

^$^The change of CA19-9 level after treatment was evaluated only in the patients with elevated baseline CA19-9 levels, the number of whom were 159 in the total NPS-treated patients and 102 in the NPS-treated patients without progression within 4 cycles of treatment.

^**^The best response to NPS chemotherapy was CR, PR, or SD in the NPS-treated patients.

CA19-9, Carbohydrate Antigen 199; CI, confidence interval; CR, complete response; ECOG PS, Eastern Cooperative Oncology Group Performance Status; HR, hazard ratio; NA, not applicable; NPS, nab-paclitaxel plus S-1; ORR, objective response rate; OS, overall survival; PFS, progression free survival; PR, partial response; SD, stable disease.

### Overall and Stratified Survival Analyses

Survival of all the NPS-treated patients and those without progression within 4 months during first-line NPS treatment are illustrated in **Figure S1**; the mPFS were 6.0 and 8.0 months, respectively, and the mOS 11.2 and 14.3 months, respectively (**Table 1**).

**Figure 2:**
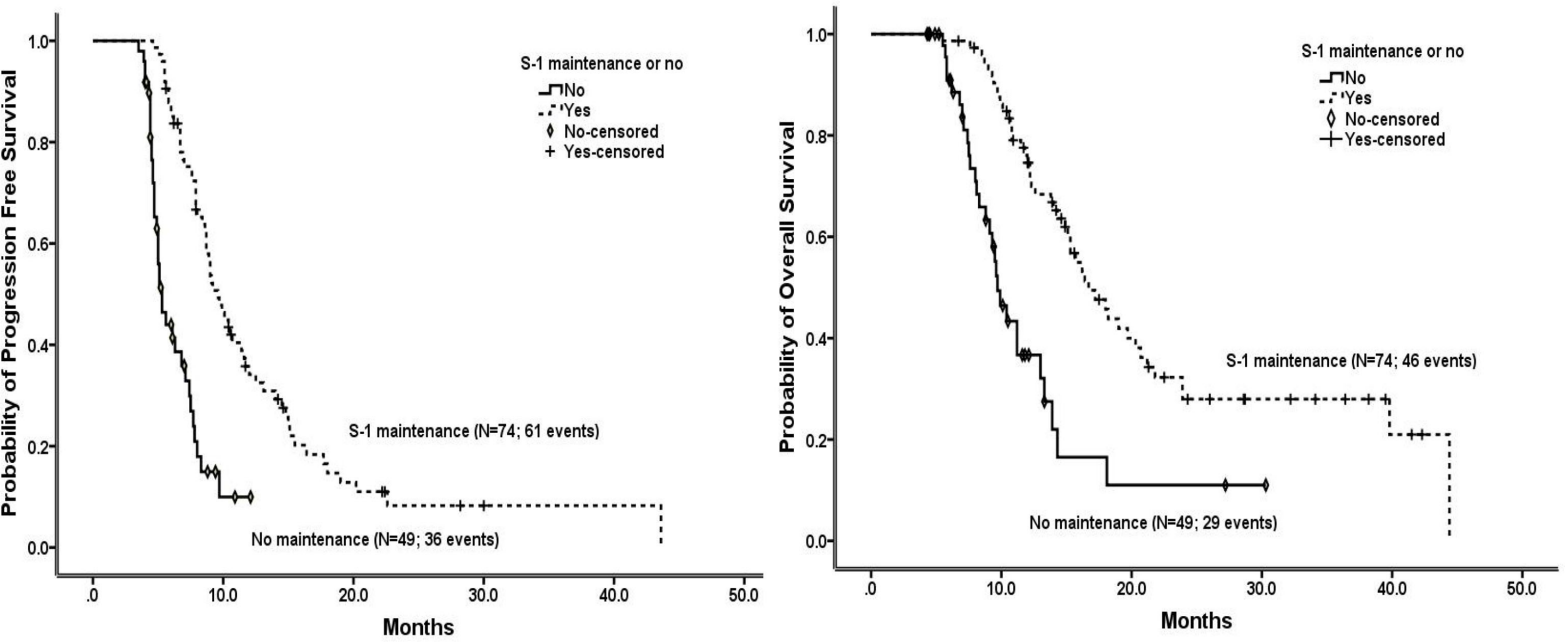
Progression-free survival (left) and overall survival (right) in the patients without progression within 4 months during NPS chemotherapy who received or not received S-1 maintenance therapy, estimated using the Kaplan-Meier method.

The survival of patients without progression within 4 months during NPS chemotherapy who received S-1 maintenance therapy, overall and in subgroups stratified by patient and tumor characteristics, are shown in **Figure 2** and **Tables 1** and **S1**. Overall, the median PFS and OS were 9.6 and 16.7 months, respectively, in the S-1 maintenance group, and the 1-year OS rate was 74.6%. Among patients with S-1 maintenance, the median OS was as long as 39.8 months in cases with locally advanced disease and 20.7 months in those with ECOG score of 0, and the 1-year OS was as high as 86.8% in cases <58 years and 82.5% in those with ECOG score of 0.

### Prognostic Factors

In all patients treated with first-line NPS chemotherapy, demographic, clinical, and pathological factors associated with survival are shown in **Table S2**. After multivariable adjustment, patients with ECOG score of 1 (HR_PFS_=1.63; HR_OS_=2.77), with base-line CA19-9 levels ≥2000 U/mL (HR_PFS_=2.00; HR_OS_=1.70), and with stable disease (versus partial or complete response: HR_PFS_=2.16; HR_OS_=2.33) or progressive disease (HR_PFS_=55.00; HR_OS_=6.86) as best response to chemotherapy had significantly worse survival. Cases not having CA19-9 response had significantly worse PFS (HR_PFS_=1.89), and metastatic disease was significantly associated with worse OS (HR_OS_=2.58).

In patients without progression within 4 months during first-line NPS chemotherapy, factors associated with OS and PFS are shown in **Tables 2** and **S3**, respectively. After multivariable adjustment, ECOG score of 1 was significantly associated with worse survival (HR_OS_=6.29; HR_PFS_=3.62). Metastatic disease was significantly associated with worse OS compared to locally advanced disease (HR_OS_=2.65), and metastasis to other sites with better PFS compared to metastasis to liver only (HR_PFS_=0.34).

**Table 2:** Associations of demographic, clinical, and pathological factors with OS in overall patients without progression within 4 months during first-line NPS chemotherapy and those further receiving S-1 maintenance, estimated using the Cox proportional hazards regression.

Variable	Patients without progression within 4 months during NPS treatment			
	Overall, n=123		With S-1 maintenance, n=74	
	Multivariable HR^*^ (95% CI)	*P* value	Multivariable HR^*^ (95% CI)	*P* value
				
**S-1 maintenance treatment**		**<0.001**	NA	
Yes	0.18 (0.10-0.35)			
No	1 (ref.)			
**Age (years)**		0.624		0.066
<58	1 (ref.)		1 (ref.)	
≥58	1.13 (0.70-1.83)		1.90 (0.96-3.76)	
**Sex**		0.686		0.436
Male	1 (ref.)		1 (ref.)	
Female	1.11 (0.68-1.81)		0.77 (0.40-1.48)	
**ECOG PS score**		**<0.001**		**<0.001**
0	1 (ref.)		1 (ref.)	
1	6.29 (3.42-11.57)		5.27 (2.39-11.60)	
**Stage **		**0.049**		**0.035**
Locally advanced	1 (ref.)		1 (ref.)	
Metastatic	2.65 (1.01-7.01)		4.47 (1.11-18.01)	
**Location of primary tumor**		0.550		0.128
Head/neck	1 (ref.)		1 (ref.)	
Body/tail	0.85 (0.49-1.46)		0.57 (0.28-1.17)	
**Tumor differentiation**		0.689		0.618
Well/well-moderately/moderately differentiated	1 (ref.)		1 (ref.)	
Moderately-poorly/poorly differentiated	0.90 (0.54-1.51)		0.83 (0.39-1.75)	
**Metastasis site** ^&^		0.328		0.591
Liver only	1 (ref.)		1 (ref.)	
Liver and others	0.44 (0.12-1.67)	0.226	0.48 (0.08-2.72)	0.406
Others except liver	0.74 (0.24-2.30)	0.596	0.76 (0.18-3.26)	0.715
**Number of metastases**		0.476		0.154
0-1	1 (ref.)		1 (ref.)	
2	1.37 (0.78-2.42)	0.271	1.34 (0.62-2.90)	0.456
≥3	1.37 (0.68-2.79)	0.382	2.63 (0.99-6.99)	0.053
**Baseline CA19-9 levels**		0.845		0.586
<2000 U/mL	1 (ref.)		1 (ref.)	
≥2000 U/mL	0.95 (0.55-1.62)		1.25 (0.56-2.79)	
**>50% decline from baseline CA19-9 level** ^$^		0.538		0.493
Yes	1 (ref.)		1 (ref.)	
No	0.77 (0.34-1.76)		0.55 (0.10-3.00)	
**Best response to chemotherapy** ^**^		0.439		**0.048**
CR or PR	1 (ref.)		1 (ref.)	
SD	1.25 (0.71-2.19)		2.51 (1.01-6.22)	

^*^The multivariable HRs were calculated using the main COX proportional hazards regression model, with adjustment for age, sex, ECOG PS, stage, primary tumor location, differentiation grade, number of metastases, baseline CA19-9 levels, and best response to chemotherapy. For metastatic site (only available for metastatic diseases) and >50% decline from baseline CA19-9 level (evaluated only in patients with elevated baseline CA19-9 levels), they were additionally, respectively, included into the main model when calculating HRs for them. P<0.05 was considered to indicate statistical significance. Significant P values are shown in bold.

^&^Metastasis site was evaluated only in metastatic diseases.

^$^The change of CA19-9 level after treatment was evaluated only in the patients with elevated baseline CA19-9 levels.

^**^The best response to NPS chemotherapy was CR, PR, or SD in the NPS-treated patients.

CA19-9, Carbohydrate Antigen 199; CI, confidence interval; CR, complete response; ECOG PS, Eastern Cooperative Oncology Group Performance Status; HR, hazard ratio; NA, not applicable; NPS, nab-paclitaxel plus S-1; OS, overall survival; PR, partial response; SD, stable disease.

Patterns of the multivariable associations of factors with survival in patients who further received S-1 maintenance were similar to those in patients without progression within 4 months during first-line NPS chemotherapy, with some exceptions and variations in association strengths (**Tables 2** and **S3**). Patients with an ECOG score of 1 had significantly worse survival (HR_OS_=5.27; HR_PFS_=3.42), and metastatic disease was significantly associated with worse OS compared to locally advanced disease (HR_OS_=4.47). Notably, cases with stable disease as best response to NPS chemotherapy had significantly worse survival (HR_OS_=2.51; HR_PFS_=2.17) compared to those with CR or PR as the best response. Associations with other patient and tumor factors were not significant.

### Safety Profiles

In all patients receiving NPS chemotherapy, the most common all-grade hematological and non-hematological adverse events were neutropenia (65.4%) and peripheral neurotoxicity (50.5%), respectively, and the most common ≥Grade 3 adverse events were neutropenia (25.8%) and fatigue (4.4%), respectively; among those without progression within 4 months during NPS therapy, the most common all-grade hematological and non-hematological adverse events remained neutropenia and leukopenia (both 76.4%) and peripheral neurotoxicity (60.2%), respectively, and the most common ≥Grade 3 adverse events were neutropenia (33.3%) and peripheral neurotoxicity (4.1%), respectively (**Table S4**).

In patients further receiving S-1 maintenance therapy, incidences of the most common all-grade hematological and non-hematological adverse events, neutropenia and peripheral neurotoxicity, increased to 82.4% and 66.2%, respectively, and incidences of the most common ≥Grade 3 adverse events, neutropenia and peripheral neurotoxicity, increased to 40.5% and 6.8%, respectively (**Table 3**).

**Table 3:** Safety profiles during the first-line treatment in the S-1 maintenance group.

Adverse events according to CTCAE V4.0	With S-1 maintenance, n=74, count (%)	
	All grades	Grade 3 or above
**Hematological adverse events**		
Leukopenia	59 (79.7)	22 (29.7)
Neutropenia	61 (82.4)	30 (40.5)
Thrombocytopenia	28 (37.8)	0 (0.0)
Anemia	47 (63.5)	6 (8.1)
**Non-hematological adverse events**		
Hand-foot syndrome	19 (25.7)	1 (1.4)
Nausea and vomiting	40 (54.1)	1 (1.4)
Peripheral neurotoxicity	49 (66.2)	5 (6.8)
Elevated AST/ALT	18 (24.3)	0 (0.0)
Fatigue	18 (24.3)	1 (1.4)
Oral mucositis	10 (13.5)	1 (1.4)

CTCAE V4.0, Common Terminology Criteria for Adverse Events, Version 4.0; AST, aspartate transaminase; ALT, alanine transaminase; NA, not assessable.

## Discussion

PDAC is an aggressive and devastating disease. The majority of PDAC patients have unresectable, locally advanced, or metastatic disease at diagnosis and the survival is extremely poor (15, 16). The main reason for the refractoriness of local primary PDAC is that pancreatic cancer cells are surrounded by an intense desmoplastic reaction which may create a barrier to the drugs penetrating into the tumor and the stroma has been addressed as a potential therapeutic target. The role of the stroma proportion might explain the resistance of majorly local primary PDAC to chemotherapy (17) but not that of metastatic disease. Nevertheless, the control of disease at the primary site might help to relieve the metastatic burden. Nab-paclitaxel is an innovative molecule which depletes tumor stroma through interaction between albumin and secreted acidic protein which is rich in cysteine. In 2013, the Food and Drug Administration (FDA) approved nab-paclitaxel plus gemcitabine as the standard first-line chemotherapy for metastatic pancreatic cancer based on the MPACT trial (18). However, several common and severe adverse events, such as neutropenia, thrombocytopenia, and peripheral neurotoxicity, affected the compliance and clinical application of this regimen. S-1, an oral fluoropyrimidine derivative, has been shown as a very promising treatment agent with good tolerability for PDAC (19). Addition of nab-paclitaxel to S-1 as first-line treatment has shown encouraging activity and efficacy in APAC with promising ORR and survival in our previous NASPAC study (12), which was considered as one of the standards of care in APAC therapy (20–22).

For APAC where cure is scarce, the main aim of management should be prolongation of survival and improvement of quality of life, through inhibition of cancer progression without severely compromising physical status. Although the strategy of continuing intensive chemotherapy until disease progression or for 6 months has been shown to improve the survival of metastatic PDAC based on two famous phase III trials (18, 23), recently increasing evidence seems to favor the maintenance strategy for APAC (8, 24–26). Following our previous phase II trial on NPS for APAC where part of the patients further received S-1 maintenance and showed good prognosis (12), another single-arm phase II trial also showed that NPS regimen as first-line therapy followed by S-1 maintenance for APAC presented encouraging ORR and survival and manageable toxicities (27). Herein we further described the characteristics, tumor responses, survival, and safety profiles of patients receiving subsequent S-1 maintenance therapy, and explored prognostic factors in cases further having S-1 maintenance.

We observed that the ORR and the mOS in the total 182 patients with APAC receiving NPS (ORR, 53.8%; mOS, 11.2 months) were similar with those reported in our previous phase II NASPAC trial (12) (ORR, 50.0%; mOS, 9.4 months), and in another study on NPS followed by S-1 maintenance for APAC (ORR, 53.1%; mOS, 11.2 months) (27), suggesting the good verifiability of the efficacy of the NPS regimen. In this study, 123 (67.6%) patients without progression within the first 4 months during NPS treatment, of whom 74 patients received S-1 maintenance according to the physicians’ discretion based on the patients’ preference and ECOG performance status as well as on evaluations of efficacies and benefits. However, it is worthwhile to suggest that it might be better to change the strategy of APAC treatment from continuous intensive chemotherapy to the single-agent maintenance mode at a proper time-point.

Favorable outcomes remain challenging in PDAC, in part due to the lack of validated markers for patient and treatment selection and thus optimal clinical decision-making. Increasingly, however, therapeutic advancement for PDAC is accompanied by evaluations of response markers (28). Tumor biological behavior was considered as an important prognostic factor. In borderline resectable PDAC, patients with initially elevated CA19-9 levels, who do not have a decline to a sustained low level in response to chemotherapy, are at risk for disease progression and poor survival (29), which highlight the importance of CA19-9 response in achieving satisfactory ORR and favorable survival. In addition, tumor burden was a widely recognized prognostic factor in many kinds of cancers including pancreatic cancer. We could speculate that the maintenance strategy might not work well if the tumor burden was too large and that best supportive care instead of continuous chemotherapy or sequential treatment with two different chemotherapy regimens might be a better option.

Our previous phase II trial showed that in APAC patients receiving NPS chemotherapy, remarkable survival benefits were observed in female patients (mPFS: 7.7 months, mOS: 18.2 months) with CA19-9 response (mPFS: 6.8 months, mOS: 11.8 months), objective response (mPFS: 6.9 months, mOS: 12.2 months), or baseline ECOG score of 0 (mPFS: 7.5 months, mOS: 16.1 months) (12). Herein, we further found that only stable disease best responded to chemotherapy, with an ECOG score of 1, and metastatic disease significantly and independently predicted worse survival in patients receiving S-1 maintenance after NPS chemotherapy.

In patients receiving S-1 maintenance after first-line NPS therapy, the most common all-grade and Grade 3 or above hematological adverse events were neutropenia (82.4% and 40.5%, respectively), and the most common all-grade and Grade 3 or above non-hematological adverse events were peripheral neurotoxicity (66.2% and 6.8%, respectively). S-1 maintenance was significantly associated with a 17.5% unit increase in all-grade hand-foot syndrome incidence and 18.1% increase in Grade 3 or above neutropenia incidence. The incidence of Grade 3 or above neutropenia was higher in our study than in a previous phase II study with similar research focus (27.6%) (27), which could be due to the differences in study protocol and patient and tumor characteristics. Also considering the higher ORR and survival in our study, there may be room for modification in the chemotherapy regimen with further optimized and well-balanced oncologic benefits and adverse events. The prognostic role of treatment-related peripheral neuropathy was limited during nab-paclitaxel plus gemcitabine treatment for patients with metastatic pancreatic cancer (30), and the replace of gemcitabine with S-1 is expected to be associated with safer profiles. While generally manageable, various adverse events during treatment call for meticulous and specific monitoring.

There are several important points to further discuss. Pancreas body/tail cancers comprised the majority in our study, where only locally advanced or metastatic unresectable pancreatic cancers were included. This site distribution might not be in compliance with the distribution in overall pancreatic cancers, as pancreatic body/tail cancers might more often involve major vasculatures rendering them unresectable and might be more insidious in disease course without causing obvious symptoms or signs, like jaundice, at the early stage of disease, making them more often diagnosed at an advanced stage. In contrast, pancreatic head/neck cancers might be more often detected and surgically managed at an earlier state as they are relatively more distant from major vasculatures and more often surgically manageable, having more often symptoms and/or signs (especially obstructive jaundice) at an earlier stage (2). The relatively small case number could be another reason.

S-1 has been majorly used in Asian countries based on some major clinical trials conducted in Asia (31, 32). The S-1 agent has been shown to potentially improve the efficacy of oral fluorinated pyrimidines by combining the antineoplastic agent with two biologic modulators aimed at reducing bowel toxicity and increasing the antitumor efficacy of the fluoro-pyrimidine. Patient and cancer characteristics might differ across different ethnicities, with possibly different efficacies and/or safety profiles of S-1 treatment. The outcomes based on Asian patients might not be directly generalizable or applicable to Western patients before further relevant evidence in the Western population is revealed, as with the case in gastric cancer (33). In Western populations tolerable doses of S-1 may be lower as a result of higher folate levels and genetic differences in drug metabolism between Western and Asian populations (33).

In our real-world study, some patients with stable disease within a certain time period during NPS treatment received subsequent S-1 monotherapy as maintenance. For patients with progressive pancreatic cancers not responsive to induction combination NPS chemotherapy including S-1, S-1 monotherapy, as maintenance, might not help to prevent disease from further progression. For patients with stable disease rather than progressive disease during induction chemotherapy, the regimen components in S-1 might help to inhibit cancer progression to some extent. Notably, the combination NPS regimen, especially the nab-paclitaxel component, might cause severe adverse events including peripheral neurotoxicity and neutropenia, which significantly deteriorates patients’ quality of life; and the toxicities might accumulate with longer duration of use. It is uncertain whether the possibly limited survival benefits brought about by the addition of nab-paclitaxel to S-1 might outweigh or be compromised by the toxicities caused, especially in the longer term. S-1, an oral fluoropyrimidine derivative, has been shown to have good efficacy and tolerability with milder adverse events (31, 32).

Our study had some limitations. Metastatic and locally advanced pancreatic cancers were both included in this study, which might present different prognostic profiles. However, S-1 maintenance followed NPS showed good compliance and efficacy in both metastatic and locally advanced diseases. Compared to other chemotherapeutic drugs, S-1 is more easily administrable with better health economic indices. Before further randomized evidence is obtained, our analysis, based on the previous phase II trial and with careful and rigorous statistics and interpretations, provides important evidence supporting S-1 maintenance following NPS chemotherapy for APAC, thus laying key foundations for further endeavors and offering new hope for this disease of despair.

In conclusion, S-1 maintenance after tumor response or stable disease induced by first-line NPS treatment was effective and well-tolerated for some patients with APAC, which offers a promising alternative treatment strategy with encouraging survival for APAC. Further relevant investigations with larger sample sizes and with randomized design are warranted to validate our findings.

## Data Availability Statement

The datasets presented in this article are not readily available because the data were used under license for the current study. Requests to access the datasets should be directed to the corresponding author.

## Ethics Statement

The studies involving human participants were reviewed and approved by the institutional review boards at the Chinese PLA General Hospital, Beijing, China. The patients/participants provided their written informed consent to participate in this study.

## Author Contributions

GD and YS conceived and designed the study. YS, HY, YL, SG, LH, QH, and ZW collected the original data. JY and JL contributed to blinded pathological review. YS, HY, YL, LH, QH, and GD analyzed and interpreted the data. YS drafted the paper. All authors participated in revising the manuscript critically. All authors read and approved the final manuscript.

## Funding

This work was supported by Suzhou collaborative medicine foundation BG-BJ-3752. The funding body has no role in design and conduct of the study; collection, management, analysis, and interpretation of the data; and preparation, review, or approval of the manuscript.

## Conflict of Interest

The authors declare that the research was conducted in the absence of any commercial or financial relationships that could be construed as a potential conflict of interest.

## Publisher’s Note

All claims expressed in this article are solely those of the authors and do not necessarily represent those of their affiliated organizations, or those of the publisher, the editors and the reviewers. Any product that may be evaluated in this article, or claim that may be made by its manufacturer, is not guaranteed or endorsed by the publisher.

## Supplementary Material

Supplementary Figure 1 Progression-free survival (left) and overall survival (right) in the whole NPS-treated population and in the population without progression within 4 months during first-line NPS therapy, estimated using the Kaplan-Meier method.

## Abbreviations

CI, confidence interval; PDAC, pancreatic ductal adenocarcinoma; HR, hazard ratio.
