# Supplementary material for: S-1 Maintenance Therapy After First-Line Treatment With Nab-Paclitaxel Plus S-1 for Advanced Pancreatic Adenocarcinoma: A Real-World Study
Source: Front Oncol. 2022 May 13;12:865404. doi: 10.3389/fonc.2022.865404 (PMC9141286; doi:10.3389/fonc.2022.865404)

Figure S1. Progression-free survival (left) and overall survival (right) in the whole NPS-treated population and in the population without progression within 4 months during first-line NPS therapy, estimated using the Kaplan-Meier method.

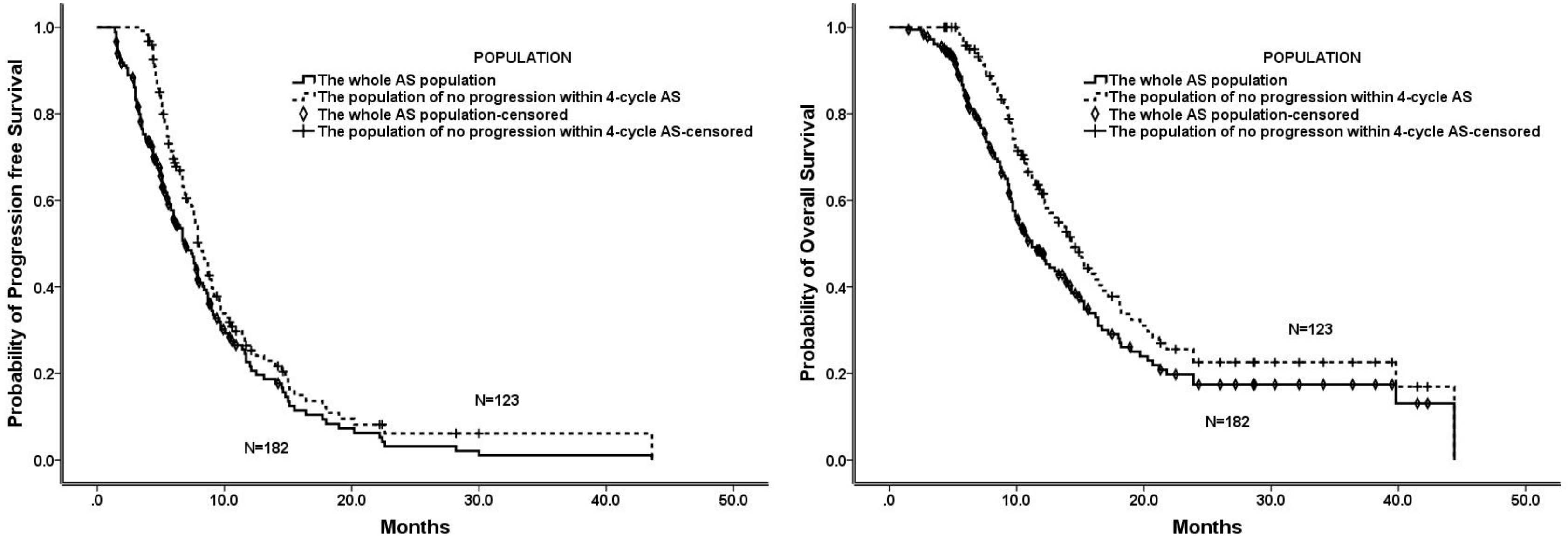

Supplement: Supplementary Figure 1 — Progression-free survival (left) and overall survival (right) in the whole NPS-treated population and in the population without progression within 4 months during first-line NPS therapy, estimated using the Kaplan-Meier method. [file Image_1.pdf]
