# Supplementary material for: S-1 Maintenance Therapy After First-Line Treatment With Nab-Paclitaxel Plus S-1 for Advanced Pancreatic Adenocarcinoma: A Real-World Study
Source: Front Oncol. 2022 May 13;12:865404. doi: 10.3389/fonc.2022.865404 (PMC9141286; doi:10.3389/fonc.2022.865404)
Supplement: Supplementary file 2 [file Table_1.docx]

**Table S1. Overall survival of patients receiving S-1 maintenance therapy in the population without progression within 4 months NPS chemotherapy, according to pre-treatment factors and treatment response**

| **Subgroup** | **S-1 maintenance** | | |
| --- | --- | --- | --- |
|  | **Event/n (%)** | **median OS** | **1-year OS (%)** |
| **Overall** | 46/74 (62.2) | 16.7 | 74.6 |
| **Age (years)** |  |  |  |
| <58 | 16/33 (48.5) | 19.7 | 86.8 |
| ≥58 | 30/41 (73.2) | 14.3 | 59.0 |
| **Sex** |  |  |  |
| Male | 22/37 (59.5) | 16.2 | 70.9 |
| Female | 24/37 (64.9) | 18.2 | 71.9 |
| **ECOG PS score** |  |  |  |
| 0 | 27/55 (49.5) | 20.7 | 82.5 |
| 1 | 19/19 (100.0) | 12.0 | 47.4 |
| **Stage** |  |  |  |
| Locally advanced | 3/9 (33.3) | 39.8 | 38.9 |
| Metastatic | 43/65 (66.2) | 16.2 | 74.3 |
| **Location of primary tumor** |  |  |  |
| Head/neck | 17/26 (65.4) | 16.4 | 69.0 |
| Body/tail | 29/48 (60.4) | 17.2 | 72.7 |
| **Tumor differentiation** |  |  |  |
| Well/well-moderately/moderately differentiated | 22/35 (62.9) | 16.7 | 70.9 |
| Moderately-poorly/poorly differentiated | 24/39 (61.5) | 17.2 | 72.0 |
| **Metastasis site** |  |  |  |
| Liver | 12/20 (60.0) | 16.4 | 60.6 |
| Liver and others | 18/25 (72.0) | 14.0 | 66.9 |
| Others except liver | 13/20 (62.1) | 18.0 | 72.4 |
| **Number of** **metastases** |  |  |  |
| 0-1 | 18/35 (51.4) | 19.7 | 69.2 |
| 2 | 18/27 (66.7) | 14.9 | 80.6 |
| ≥3 | 10/12 (83.3) | 16.2 | 57.1 |
| **Baseline CA19-9 levels** |  |  |  |
| <2000 U/mL | 28/52 (53.8) | 20.3 | 75.6 |
| ≥2000 U/mL | 18/22 (81.8) | 15.3 | 61.9 |
| **Baseline tumor biomarkers** |  |  |  |
| Elevated level of CA19-9 | 6/16 (37.5) | 39.8 | 77.4 |
| Elevated levels of CA19-9 and others | 30/43 (69.8) | 15.7 | 72.8 |
| Elevated levels of others except CA19-9 | 8/13 (61.5) | 14.0 | 52.7 |
| **Best response to chemotherapy** |  |  |  |
| PR or CR | 36/57 (63.2) | 18.2 | 76.6 |
| SD | 10/17 (58.8) | 13.8 | 52.1 |

^*^The median OS and the 1-year OS rate of the S-1 maintenance group were estimated using the Kaplan-Meier method.

NPS, nab-paclitaxel plus S-1; ECOG PS, Eastern Cooperative Oncology Group Performance Status; CA19-9, Carbohydrate Antigen 199; CR, complete response; PR, partial response; SD, stable disease; PD, progressive disease; OS, overall survival.
