# Supplementary material for: S-1 Maintenance Therapy After First-Line Treatment With Nab-Paclitaxel Plus S-1 for Advanced Pancreatic Adenocarcinoma: A Real-World Study
Source: Front Oncol. 2022 May 13;12:865404. doi: 10.3389/fonc.2022.865404 (PMC9141286; doi:10.3389/fonc.2022.865404)
Supplement: Supplementary file 3 [file Table_2.docx]

**Table S2. Associations of demographic, clinical, and pathological factors with PFS and OS in all the patients with advanced pancreatic cancer treated with first-line NPS chemotherapy, estimated using the Cox proportional hazards regression**

| **Variable** | **PFS** | | | | **OS** | | | |
| --- | --- | --- | --- | --- | --- | --- | --- | --- |
|  | **Univariable HR (95% CI)** | ***P* value** | **Multivariable**  **HR**^*^ **(95% CI)** | ***P* value** | **Univariable HR (95% CI)** | ***P* value** | **Multivariable HR**^*^ **(95% CI)** | ***P* value** |
| **Age (years)** |  | 0.715 |  | 0.062 |  | 0.365 |  | 0.543 |
| <58, n=91 | 1 (ref.) |  | 1 (ref.) |  | 1 (ref.) |  | 1 (ref.) |  |
| ≥58, n=91 | 0.94 (0.68-1.30) |  | 0.72 (0.51-1.02) |  | 1.19 (0.82-1.71) |  | 1.13 (0.77-1.66) |  |
| **Sex** |  | **0.017** |  | 0.070 |  | **0.049** |  | 0.065 |
| Male, n=108 | 1 (ref.) |  | 1 (ref.) |  | 1 (ref.) |  | 1 (ref.) |  |
| Female, n=74 | 0.67 (0.48-0.93) |  | 0.72 (0.51-1.03) |  | 0.68 (0.47-1.00) |  | 0.69 (0.47-1.02) |  |
| **ECOG PS score** |  | **0.004** |  | **0.020** |  | **<0.001** |  | **<0.001** |
| 0, n=142 | 1 (ref.) |  | 1 (ref.) |  | 1 (ref.) |  | 1 (ref.) |  |
| 1, n=40 | 1.72 (1.18-2.50) |  | 1.63 (1.08-2.46) |  | 2.20 (1.48-3.27) |  | 2.77 (1.79-4.28) |  |
| **Stage** |  | **0.010** |  | 0.127 |  | **0.009** |  | **0.036** |
| Locally advanced, n=15 | 1 (ref.) |  | 1 (ref.) |  | 1 (ref.) |  | 1 (ref.) |  |
| Metastatic, n=167 | 2.35 (1.23-4.47) |  | 1.71 (0.86-3.38) |  | 3.04 (1.33-6.97) |  | 2.58 (1.06-6.27) |  |
| **Location of primary tumor** |  | 0.728 |  | 0.761 |  | 0.596 |  | 0.086 |
| Head/neck, n=62 | 1 (ref.) |  | 1 (ref.) |  | 1 (ref.) |  | 1 (ref.) |  |
| Body/tail, n=120 | 1.06 (0.76-1.49) |  | 0.94 (0.64-1.38) |  | 0.90 (0.62-1.32) |  | 0.71 (0.47-1.05) |  |
| **Tumor differentiation** |  | 0.096 |  | 0.215 |  | 0.174 |  | 0.373 |
| Well/well-moderately/moderately differentiated, n=67 | 1 (ref.) |  | 1 (ref.) |  | 1 (ref.) |  | 1 (ref.) |  |
| Moderately-poorly/poorly differentiated, n=115 | 1.33 (0.95-1.86) |  | 1.26 (0.88-1.80) |  | 1.30 (0.89-1.91) |  | 1.21 (0.80-1.82) |  |
| **Metastasis site** |  | **0.009** |  | 0.247 |  | 0.232 |  | 0.844 |
| Liver, n=66 | 1 (ref.) |  | 1 (ref.) |  | 1 (ref.) |  | 1 (ref.) |  |
| Liver and others, n=66 | 0.94 (0.65-1.35) | 0.721 | 0.69 (0.26-1.83) | 0.135 | 1.11 (0.73-1.69) | 0.632 | 0.74 (0.24-2.34) | 0.610 |
| Others except liver, n=35 | 0.50 (0.31-0.79) | **0.003** | 0.54 (0.24-1.21) | 0.452 | 0.71 (0.42-1.20) | 0.200 | 0.75 (0.29-1.98) | 0.562 |
| **Number of** **metastases** |  | 0.105 |  | 0.279 |  | 0.056 |  | 0.205 |
| 0-1, n=92 | 1 (ref.) |  | 1 (ref.) |  | 1 (ref.) |  | 1 (ref.) |  |
| 2, n=53 | 1.03 (0.71-1.50) | 0.869 | 1.06 (0.70-1.59) | 0.791 | 1.24 (0.80-1.91) | 0.333 | 1.11 (0.70-1.76) | 0.667 |
| ≥3, n=37 | 1.55 (1.02-2.35) | **0.040** | 1.44 (0.91-2.27) | 0.116 | 1.76 (1.11,2.79) | **0.016** | 1.57 (0.95-2.58) | 0.077 |
| **Baseline CA19-9 levels** |  | **<0.001** |  | **<0.001** |  | **<0.001** |  | **0.010** |
| <2000 U/mL, n=94 | 1 (ref.) |  | 1 (ref.) |  | 1 (ref.) |  | 1 (ref.) |  |
| ≥2000 U/mL, n=88 | 2.02 (1.45-2.81) |  | 2.00 (1.40-2.87) |  | 2.03 (1.40-2.95) |  | 1.70 (1.14-2.55) |  |
| **>50% decline from baseline CA19-9 level** | | **<0.001** |  | **0.008** |  | **0.004** |  | 0.187 |
| Yes, n=94 | 1 (ref.) |  | 1 (ref.) |  | 1 (ref.) |  | 1 (ref.) |  |
| No, n=65 | 2.56 (1.78-3.68) |  | 1.89 (1.19-3.02) |  | 1.84 (1.22-2.77) |  | 1.40 (0.85-2.29) |  |
| **Best response to chemotherapy** | | **<0.001** |  | **<0.001** |  | **<0.001** |  | **<0.001** |
| PR or CR, n=98 | 1 (ref.) |  | 1 (ref.) |  | 1 (ref.) |  | 1 (ref.) |  |
| SD, n=67 | 1.98 (1.38-2.83) | **<0.001** | 2.16 (1.49-3.14) | **<0.001** | 2.04 (1.36-3.07) | **0.001** | 2.33 (1.52-3.58) | **<0.001** |
| PD, n=17 | 48.73 (24.09-98.57) | **<0.001** | 55.00 (24.93-121.35) | **<0.001** | 5.52 (2.72-11.20) | **<0.001** | 6.86 (3.19-14.77) | **<0.001** |

^*^The multivariable HRs were calculated using the COX proportional hazards regression, with adjustment for age, sex, ECOG PS, stage, primary tumor location, differentiation grade, number of metastases, baselineCA19-9 levels, and best response to chemotherapy. For metastatic site (available for 167 (92%) metastatic diseases) and >50% decline from baseline CA19-9 level (missing for 23 cases (13%)), they were additionally respectively included into the main model when calculating HRs for them. *P*<0.05 was considered to indicate statistical significance. Significant *P* values are shown in bold.

CA19-9, Carbohydrate Antigen 199; CI, confidence interval; CR, complete response; ECOG PS, Eastern Cooperative Oncology Group Performance Status; HR, hazard ratio; NPS, nab-paclitaxel plus S-1; OS, overall survival; PFS, progression free survival; PR, partial response; SD, stable disease.
