# Supplementary material for: S-1 Maintenance Therapy After First-Line Treatment With Nab-Paclitaxel Plus S-1 for Advanced Pancreatic Adenocarcinoma: A Real-World Study
Source: Front Oncol. 2022 May 13;12:865404. doi: 10.3389/fonc.2022.865404 (PMC9141286; doi:10.3389/fonc.2022.865404)
Supplement: Supplementary file 4 [file Table_3.docx]

**Table S3. Associations of demographic, clinical, and pathological factors with PFS in overall patients without progression within 4 months during first-line NPS chemotherapy and those further receiving S-1 maintenance, estimated using the Cox proportional hazards regression**

| **Variable** | **Patients without progression within 4 months during NPS treatment** | | | | |
| --- | --- | --- | --- | --- | --- |
|  | **Overall, n=123** | |  | **With S-1 maintenance, n=74** | |
|  | **Multivariable HR**^*^ **(95% CI)** | ***P* value** |  | **Multivariable HR**^*^ **(95% CI)** | ***P* value** |
| **S-1 maintenance treatment** |  | **<0.001** |  | NA |  |
| Yes | 0.16 (0.09-0.29) |  |  |  |  |
| No | 1 (ref.) |  |  |  |  |
| **Age (years)** |  | 0.990 |  |  | 0.784 |
| <58 | 1 (ref.) |  |  | 1 (ref.) |  |
| ≥58 | 1.00 (0.65-1.53) |  |  | 1.09 (0.60-1.95) |  |
| **Sex** |  | 0.416 |  |  | 0.094 |
| Male | 1 (ref.) |  |  | 1 (ref.) |  |
| Female | 0.84 (0.54-1.29) |  |  | 0.63 (0.36-1.08) |  |
| **ECOG PS score** |  | **<0.001** |  |  | **0.001** |
| 0 | 1 (ref.) |  |  | 1 (ref.) |  |
| 1 | 3.62 (2.07-6.35) |  |  | 3.42 (1.67-7.00) |  |
| **Stage** |  | 0.182 |  |  | 0.563 |
| Locally advanced | 1 (ref.) |  |  | 1 (ref.) |  |
| Metastatic | 1.63 (0.80-3.32) |  |  | 1.30 (0.54-3.10) |  |
| **Location of primary tumor** |  | 0.496 |  |  | 0.988 |
| Head/neck | 1 (ref.) |  |  | 1 (ref.) |  |
| Body/tail | 0.84 (0.52-1.38) |  |  | 1.00 (0.57-1.79) |  |
| **Tumor differentiation** |  | 0.725 |  |  | 0.941 |
| Well/well-moderately/moderately differentiated | 1 (ref.) |  |  | 1 (ref.) |  |
| Moderately-poorly/poorly differentiated | 1.08 (0.71-1.65) |  |  | 1.02 (0.58-1.81) |  |
| **Metastasis site**^&^ |  | 0.074 |  |  | 0.368 |
| Liver only | 1 (ref.) |  |  | 1 (ref.) |  |
| Liver and others | 0.37 (0.12-1.14) | 0.083 |  | 0.46 (0.11-1.98) | 0.296 |
| Others except liver | 0.34 (0.13-0.86) | **0.022** |  | 0.44 (0.14-1.38) | 0.159 |
| **Number of** **metastases** |  | 0.073 |  |  | 0.350 |
| 0-1 | 1 (ref.) |  |  | 1 (ref.) |  |
| 2 | 1.53 (0.92-2.56) | 0.103 |  | 0.87 (0.46-1.63) | 0.665 |
| ≥3 | 1.69 (0.93-3.06) | 0.084 |  | 1.71 (0.73-4.01) | 0.217 |
| **Baseline CA19-9 levels** |  | 0.864 |  |  | 0.699 |
| <2000 U/mL | 1 (ref.) |  |  | 1 (ref.) |  |
| ≥2000 U/mL | 0.96 (0.59-1.56) |  |  | 1.15 (0.57-2.32) |  |
| **>50% decline from baseline CA19-9 level**^$^ |  | 0.099 |  |  | 0.511 |
| Yes | 1 (ref.) |  |  | 1 (ref.) |  |
| No | 0.52 (0.24-1.13) |  |  | 3.03 (0.87-10.65) |  |
| **Best response to chemotherapy**^**^ |  | 0.800 |  |  | **0.029** |
| CR or PR | 1 (ref.) |  |  | 1 (ref.) |  |
| SD | 1.07 (0.65-1.76) |  |  | 2.17 (1.09-4.35) |  |

^*^The multivariable HRs were calculated using the main COX proportional hazards regression model, with adjustment for age, sex, ECOG PS, stage, primary tumor location, differentiation grade, number of metastases, baseline CA19-9 levels, and best response to chemotherapy. For metastatic site (only available for metastatic diseases) and >50% decline from baseline CA19-9 level (evaluated only in patients with elevated baseline CA19-9 levels), they were additionally respectively included into the main model when calculating HRs for them. *P*<0.05 was considered to indicate statistical significance. Significant *P* values are shown in bold.

^&^Metastasis site was evaluated only in metastatic diseases.

^$^The change of CA19-9 level after treatment was evaluated only in the patients with elevated baseline CA19-9 levels.

^**^The best response to NPS chemotherapy was CR, PR, or SD in the NPS-treated patients.

CA19-9, Carbohydrate Antigen 199; CI, confidence interval; CR, complete response; ECOG PS, Eastern Cooperative Oncology Group Performance Status; HR, hazard ratio; NA, not applicable; NPS, nab-paclitaxel plus S-1; PFS, progression free survival; PR, partial response; SD, stable disease.
