# Supplementary material for: S-1 Maintenance Therapy After First-Line Treatment With Nab-Paclitaxel Plus S-1 for Advanced Pancreatic Adenocarcinoma: A Real-World Study
Source: Front Oncol. 2022 May 13;12:865404. doi: 10.3389/fonc.2022.865404 (PMC9141286; doi:10.3389/fonc.2022.865404)
Supplement: Supplementary file 5 [file Table_4.docx]

**Table S4. Safety profiles in the whole population (n=182) and the population without progression during the first 4 cycles of NPS treatment (n=123)**

| **Adverse events according to CTCAE V4.0** | **All grades** | | **Grade 3 or above** | |
| --- | --- | --- | --- | --- |
|  | **Whole, count (%)** | **Without progression during the first 4 cycles of NPS therapy, count (%)** | **Whole, count (%)** | **Without progression during the first 4 cycles of NPS therapy, count (%)** |
| **Hematological adverse events** | |  |  |  |
| Leukopenia | 117 (64.3) | 94 (76.4) | 38 (20.9) | 33 (26.8) |
| Neutropenia | 119 (65.4) | 94 (76.4) | 47 (25.8) | 41 (33.3) |
| Thrombocytopenia | 55 (30.2) | 44 (35.8) | 2 (1.1) | 0 (0.0) |
| Anemia | 103 (56.6) | 75 (61.0) | 10 (5.5) | 8 (6.5) |
| **Non-hematological adverse events** | |  |  |  |
| Hand-foot syndrome | 30 (16.5) | 23 (18.7) | 1 (0.5) | 1 (0.8) |
| Nausea and vomiting | 77 (42.3) | 60 (48.8) | 3 (1.6) | 1 (0.8) |
| Peripheral neurotoxicity | 92 (50.5) | 74 (60.2) | 7 (3.8) | 5 (4.1) |
| Elevated AST/ALT level | 39 (21.4) | 28 (22.8) | 0 (0.0) | 0 (0.0) |
| Fatigue | 50 (27.5) | 30 (24.4) | 8 (4.4) | 3 (2.4) |
| Oral mucositis | 19 (10.4) | 17 (13.8) | 4 (2.2) | 3 (2.4) |

CTCAE V4.0, Common Terminology Criteria for Adverse Events, Version 4.0; AST, aspartate transaminase; ALT, alanine transaminase.
